# Exploring the role of dimethylarginine dimethylaminohydrolase-mediated reduction in tissue asymmetrical dimethylarginine levels in cardio-protective mechanism of ischaemic postconditioning in rats

**DOI:** 10.22038/IJBMS.2019.14067

**Published:** 2019-12

**Authors:** Kamaldeep Kaur, Nirmal Singh, R. K. Dhawan

**Affiliations:** 1 Department of Pharmaceutical Sciences and Drug Research, Punjabi University, Patiala; 2 Department of Pharmacology, Khalsa College of Pharmacy, Amritsar

**Keywords:** ADMA, DDAH, Nitric Oxide, Postconditioning, Myocardial ischaemia-reperfusion injury

## Abstract

**Objective(s):**

Reperfusion of ischaemic myocardium results in reduced nitric oxide (NO) biosynthesis by endothelial nitric oxide synthase (eNOS) leading to endothelial dysfunction and subsequent tissue damage. Impaired NO biosynthesis may be partly due to increased levels of asymmetrical dimethylarginine (ADMA), an endogenous inhibitor of eNOS. As dimethylarginine dimethylaminohydrolase (DDAH) is a key enzyme responsible for degradation of ADMA, the present study was designed to explore the role of DDAH/ADMA/NO pathway in cardio-protective mechanism of ischaemic postconditioning.

**Materials and Methods:**

Isolated rat hearts were subjected to myocardial ischaemia for 30 min followed by reperfusion for 2 hours in control group. Myocardial injury was assessed by measurement of infarct size, left ventricular developed pressure (LVDP), lactate dehydrogenase (LDH) and creatine kinase (CK) enzymes in coronary effluents. The reperfused hearts were homogenised and tissue concentration of nitrite, ADMA level and DDAH enzyme activity was determined.

**Results:**

A significant increase in infarct size, LDH, CK release in coronary effluents and ADMA level in myocardial tissue was observed in control group. The increase in tissue ADMA coincided with reductions of NO tissue concentrations and DDAH activity. Ischaemic postconditioning significantly attenuated ischaemia-reperfusion induced myocardial injury manifested in the terms of decreased infarct size, LDH, CK, tissue ADMA along with increase in NO levels and DDAH enzyme activity. Pretreatment with L-Homocysteine (300 µM), a competitive inhibitor of DDAH, and L-NG-nitroarginine methyl ester (L-NAME; 100 µM), an inhibitor of eNOS, completely abolished ischaemic postconditioning-induced myocardial protection.

**Conclusion:**

Enhancing DDAH activity by postconditioning may be a novel target to reduce ADMA level and increase NO bioavailability to prevent myocardial ischaemia-reperfusion injury.

## Introduction

Myocardial Infarction is the leading cause of death throughout the world (1). Myocardial infarction (MI) occurs due to ischemia resulting from occlusion caused by thrombus superimposed over atherosclerotic coronary artery. Early Reperfusion therapy with percutaneous trans-luminal coronary intervention or thrombolysis (2) remains the most effective treatment strategy for reducing myocardial infarct (MI) size, preserving left ventricular (LV) ejection fraction, and preventing the onset of heart failure. However, reperfusion initiates a series of harmful events including release of reactive free oxygen species and calcium overload that contributes to further cardiomyocyte necrosis and apoptosis, termed as ‘*Reperfusion injury’ *(3).

Ischaemic preconditioning (PC)(4), is a cardioprotective strategy in which repetitive brief periods of coronary artery occlusion and reperfusion applied before the prolonged lethal ischaemia confers cardioprotection against tissue damageinduced by prolonged ischaemia.Although preconditioning has been clinically successful in attenuating the pathological consequences of reperfusion during percutaneous transluminal coronary angioplasty (5), its use as a clinical cardioprotective strategy is limited by the inability to predict the onset of ischaemia. Moreover, in a multicenter, randomized trial, involving 970 patients with an acute STEMI, administration of cyclosporine (2.5 mg/kg IV) before percutaneous coronary intervention (PCI) did not reduce the incidence of the clinical outcomes and did not prevent adverse left ventricular remodeling at 1 year (6).

In patients presenting with an acute myocardial infarction (AMI), *ischaemic postconditioning* (PostC)(7), comprising of brief episodes of ischaemia and reperfusion at the onset of reperfusion just after ischaemic event, represents a promising treatment strategy that confer protection against ischaemia reperfusion injury. In several clinical studies, ‘*ischaemic postconditioning’* is applied during PCI (angioplasty)(8) and ‘*pharmacological postconditioning’*is achieved by infusion of pharmacological agents that mimic ischaemic postconditioning at the time of reperfusion such as intravenous administration of adenosine as an adjuvant to thrombolytic therapy in (AMISTAD) trials (9) as well as intracoronary administration during CABG (10). Similarly, adjunctive exenatide therapy (11)and intracoronary administration of lactated ringer solution with percutaneous coronary intervention prevented reperfusion injury in patients with STEMI (12). However, mechanisms of cardio-protective effect of post-conditioning is still not clear and there is need to find out molecular mechanisms of protective effect of post-conditioning so that more specific ways can be adapted to expand clinical utility of post-conditioning in cardiovascular diseases.

Endothelial dysfunction characterized by the loss of endothelium derived nitric oxide (NO) is one of the most critical events occurring after reperfusion (13). The decrease in NO bioavailability triggers a cascade of early pathophysiological events of reperfusion injury, such as up-regulation of adhesion molecules, leukocyte adherence to endothelial cells, transmigration of polymorphonuclear cells and subsequent tissue damage due to apoptosis (14). Impaired NO biosynthesis may be partly due to increased levels of asymmetrical dimethylarginine (ADMA), an endogenous competitive inhibitor of eNOS (15). ADMA level in plasma and left ventricle are increased following myocardial infarction (16, 17). A large number of studies have reported that elevated ADMA level is an independent risk marker for total mortality in critically ill patient and cardiovascular outcomes in all populations (18). Further, high plasma ADMA level may limit therapeutic efficacy of statins in patients with coronary artery disease (19).

Dimethylarginine dimethylaminohydrolase-1 (DDAH1) is a key enzyme responsible for degradation of asymmetric dimethylarginine (ADMA). Recently, it is reported that in DDAH-I deficient mice subjected to ischaemia reperfusion, elevated tissue concentration of ADMA was associated with reduction in bioavailability of NO (20). In another study, reduction in DDAH activity resulting in increase in ADMA level in myocardial tissue was reported during early phase of reperfusion for 2 to 4 hours. Further, overexpression of DDAH was associated with decrease in tissue ADMA level as well as reperfusion injury (14). Moreover, in non-reperfused rat hearts, inhibition of DDAH activity associated with leakage of DDAH in infarct zone has been reported following two days of Myocardial infarction (21). Thus, increase in DDAH activity or expression may be a cardioprotective strategy against reperfusion injury, but no study has explored whether ischaemic post-conditioning could confer cardioprotection through modulation of DDAH activity mediated reduction of myocardial ADMA level.

Homocysteine is a sulfur-containing amino acid that arises during methionine metabolism. Hyperhomocytenemia is associated with endothelial dysfunction and is one of the risk factor for ischaemic heart disease (22). L-Homocyteine has been reported as competitive inhibitor of DDAH *In vitro *(23). Thus, the present study was designed to investigate the role of DDAH/ADMA/NO pathway in cardioprotective mechanism of ischaemic postconditioning by employing L-Homocysteine as selective DDAH inhibitor.

## Materials and Methods

***Drugs and chemicals***

L-NG-nitroarginine methyl ester (L-NAME), L-Homocysteine, asymmetric dimethylarginine (ADMA) were purchased from Sigma-Aldrich (St Louis, MO, USA). ELISA kit for ADMA was purchased from Cayman Chemicals, USA.

***Animals***

Wistar albino rats of either sex weighing 200-300 g maintained on rat feed and tap water *ad libitum, *were housed in the departmental animal house and were provided 12 hr cycle of light and dark. The experimental protocol was approved by the Institutional Animal Ethics Committee (IAEC/KCP/2015/004) and care of the animals was carried out as per the guidelines of Committee for the Purpose of control and supervision of experiments on Animals (1753/PO/E/S/14/CPCSEA) and Ministry of Environment and Forest, Government of India.

***Perfusion of isolated rat heart and measurement of haemodynamic parameters***

Rats were heparinised about 20 min before sacrificing the animal. Briefly, rats were anesthetized by an intraperitoneal (ip) injection of 50 mg/kg Thiopental sodium. Left thoracotomy was carried out to expose the hearts and hearts rapidly excised and mounted immediately on Langendorff’s apparatus (24). The heart was enclosed in a double walled jacket and the temperature of which was maintained at 37 ^°^C by circulating hot water. Isolated heart were retrogradely perfused at constant pressure of 70 mm Hg with Kreb’sHenseleit buffer, pH 7.4, maintained at 37 ^°^C, bubbled with 95% O_2_ and 5% CO_2_. Global ischemia was produced for 30 min by blocking the inflow of Kreb’s Henseleit solution, followed by reperfusion for 120 min and coronary effluent was collected at different time intervals ie, basal (immediately after stabilization), 0 min, 5 min and 30 min after reperfusion for carrying out biochemical estimations. Coronary flow rate was also measured at different time intervals to assess the degree of injury to coronary vasculature. Two thin silver electrodes fixed at the ventricular apex and origin of aorta were employed to record ECG (BPL, MK 801, Banglore, India) for monitoring heart rate.

***Assessment of infarct size***

After 120 min reperfusion, heart was removed and kept overnight at 0 ^°^C. Frozen heart was sliced into uniform sections of 2-3 mm thickness. The slices were incubated in 1% triphenyltetrazolium chloride (TTC) at 37 ^°^C in 0.2 M Tris buffer (pH 7.4) for 20 min and the extent of myocardial infarct size was assessed (25).

***Haemodynamic parameters***

Heart rate, coronary flow rate, Left ventricular developed pressure (LVDP) and dp/dt_max_ and –dp/dt _min_ were assessed using data aquistation system along with Langendorff apparatus (AD instruments)

***Biochemical parameters***

Lactate dehydrogenase (LDH) (26) and Creatine kinase (CK) (27) were measuredin coronary effluent as per previously established methods soto assess the degree of myocardial injury.

Nitric oxide level (NO) was assessed by Griess reagent (28). Nitrite concentrations were determined from the linear standard curve established using 0–100 μM sodium nitrate. Tissue NO levels were expressed as μmol/l (29).

***Determination of DDAH enzyme activity***

DDAH activity was measured spectrophotometrically according to the method of Tain and Baylis 2007. The method is based on the rate of L-citrulline production. Heart tissue were homogenised in 100 mM sodium phosphate buffer, pH 6.5 at 4 ^°^C as 10 % w/v homogenate. Then, homogenized tissues were centrifuged at 14,000 rpm for 25 min at 4 ^°^C and supernatants were collected. Tissue homogenate was adjusted to the concentration of 20 mg/ml.100 µl supernatant was mixed with 400 µl of 1 mM ADMA in sodium phosphate buffer, pH 6.5. The mixture was incubated in water bath at 37 ^°^C for 45 minutes. After the reaction was stopped with 0.5 ml 4% sulfosalicylic acid, samples were centrifuged at 3000 g for 10 min. Then 100 µl oxime reagent (diacetyl monoxime (0.08% wt/vol) in 5% acetic acid) mixed with antipyrine/ H_2_SO_4_ (antipyrine (0.5% wt/vol) in 50% sulphuric acid) was added to 100 µl supernatant (samples). Samples were thereafter incubated in 60 ^°^C for 110 min and cooled in ice bath for 10 min. L-citrulline formation was measured at 466 nm and values were subtracted of respective blanks (without ADMA).

Standard plot was prepared as serial dilutions of L-citrulline (3.25 µM – 100 µM). Protein concentration was determined by Lowery method. DDAH activity was represented as μM L-citrulline formation/g protein/min at 37 ^°^C (30).

***Asymmetric dimethylarginine (ADMA) estimation***

ADMA level was estimated in heart homogenates using ELISA kit (Bioassay Technology Lab, Cayman Chemicals, USA). Heart tissue was rinsed in PBS (pH 6.5) to remove excess blood thoroughly and weighed before homogenization. The tissue was homogenized in PBS (pH 6.5) with a glass homogenizer on ice and freezed at -20 ^°^C. Then, centrifuged at 2000-3000 RPM for approximately 20 minutes at 4 ^°^C.

***Assay procedure***

50μl standard was added to standard well and 40μl sample to sample wells and then 10μl anti-ADMA antibody to sample wells, then 50μl streptavidin-HRP to sample wells and standard wells (Not blank control well. Then plate was covered with a sealer and shaken gently and incubated for 60 minutes at 37 ^°^C. Then the sealer was removed and the plate was washed 5 times with wash buffer. Soak wells inwash buffer for 30 sec to 1 min for each wash. Blot the plate onto paper towels or other absorbent material.

Then 50μl substrate solution A and 50μl substrate solutionB was added to each well. The plate covered with a new sealer was incubated for 10 min at 37 ^°^C at room temperature in the dark. After addition of 50 μl Stop Solution to each well,the blue color changed into yellow immediately. The optical density (OD value) of each well was determined immediately using a microplate rearder at 450 nm within 30 min.

***Haematoxylin and eosin staining***

Heart tissues preserved in 10% formalin were dehydrated in graded concentration of ethanol, immersed in xylene and the embedded in paraffin. Section of 4 μm thickness were cut and placed on slide using Baker’s mounting fluid. Paraffin wax was removed by warming the slide gently, until the wax melted and then was washed with xylene. This was followed by washing with absolute alcohol and water to hydrate the sections and stained with haematoxylin for 15 min. Stained sections were washed with water and treated with 1% acid alcohol mixture for 20 sec. Acid alcohol mixture was washed off with water and sections were counterstained with 1% aqueous solution of eosin for 2 minutes. After washing with water to remove excess of eosin, sections were dehydrated using absolute alcohol, mounted using Canada balsam were observed under microscope for histological changes.

***Preparation of haematoxylin solution***

2 g of haematoxylin was dissolved in a mixture of absolute alcohol,distilled water and glycerin 100 ml each,followed by addition of 10 ml glacial acetic acid and in excess addition of potassium sulphate.

***Preparation of 1% eosin solution***

One g of eosin was weighed and dissolved in 100 ml of distilled water.

***Preparation of acid alcohol mixture***

Acid alcohol mixture was mixed by adding 1 ml of HCL in 99 ml of 70% alcohol.

***Statistical Analysis***

Values of infarct size and nitrite level are expressed as mean±SD Statistical significance is calculated by one way analysis of variance. Student–Newman–Keul’s test and Tukey-Kramer test have been used as *Post hoc* tests for multiple comparision between groups and for comparision with control group, respectively. A value of *P<*0.005 was considered to be statistically significant.

***Experimental Protocol***

**Figure 1:**
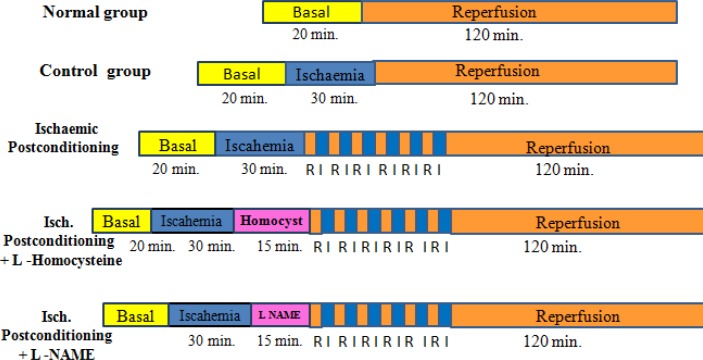
Diagrammatic representation of experimental protocol

Separate groups of animals were employed as per following protocol (Figure 1):

*Normal group*: Hearts were subjected to 20 min of equilibration 160 min of reperfusion.

*Control (I/R Injury)*: Hearts were subjected to 20 min of equilibration, followed by 30 min of global normothermic ischaemia, and 120 min of reperfusion.

*Ischaemic Postconditioning*: After 20 min of equilibration and 30 min of global normothermic ischaemia, just at the onset of reperfusion, postconditioning was induced by 6 episodes of 10 sec ischemia and 10 sec reperfusion.

*Ischaemic Postconditioning+L- Homocysteine (300 µM): *After 20 min of equilibration and 30 min ischaemia, perfusion with L–Homocyteine for 15 minutes before ischemic postconditioning.

*Ischaemic Postconditioning+L-NAME (100 µM): *After 20 min of equilibration and 30 min ischaemia, perfusion with L–NAME for 15 min before ischemic postconditioning.

## Results

***Effect on infarct size***

**Figure 2:**
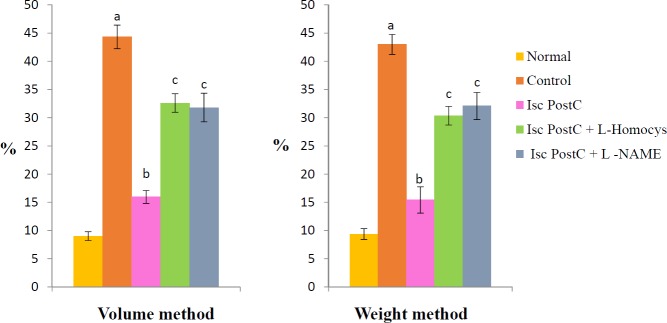
Effect of postconditioning on infarct size

Global ischaemia for 30 min followed by reperfusion for 120 min produced significant increase in myocardial infarct size calculated by volume (44.32±2.1%) and weight method (42.98±1.77%) in control group as compared to normal group (9.01±0.77% and 9.38±0.97%). Ischaemic postconditioining attenuated the increase in infarct size as compared to control group.Pretreatment with LHomocysteine (300 µM), a selective inhibitor of DDAH enzyme and LNAME (100 µM) completely abolished the ischemic postconditioning induced reduction in infarct size (Figure 2).

***Effect on Haemodynamic parameters***

Among haemodynamic parameters, there was significant decrease in heart rate (200.80±8.02 to 76.00±11.26) and coronary flow rate (8.92±0.23 to 2.90±0.34) in control group as compared to normal group. Ischaemic postconditioning significantly increased heart rate and coronary flow rate as compared to control group. Pretreatment with LHomocysteine (300 µM), a selective inhibitor of DDAH enzyme and LNAME (100 µM) completely abolished the ischaemic postconditioning induced increase in heart rate, coronary flow rate (Table 1 and 2).

No significant differences were detected in the LVDP, +dp/dtmax between groups at the end of equilibration (*P>*0.05).At the end of reperfusion, LVDP in the control group recovered to 29.0±3.9% of baseline value, whereas ischaemic postconditioned hearts showed a significantly increased recovery (LVDP 77.4±5.7%, *P<*0.05).Pretreatment with LHomocysteine (300 µM), a selective inhibitor of DDAH enzyme and LNAME (100 µM) completely abolished the ischemic postconditioning induced increase in LVDP and +dp/dtmax (Table 3 and 4).

***Effect on LDH and CK relaese***

**Figure 3:**
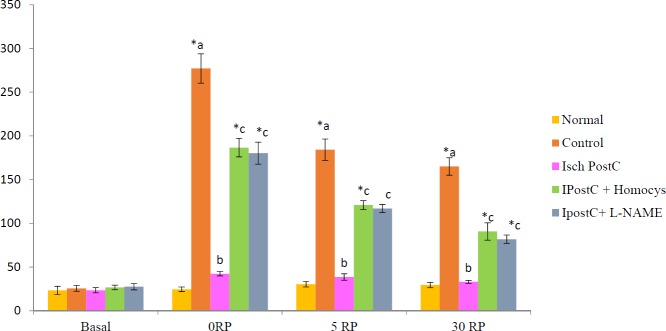
Effect of postconditioning on lactate dehydrogenase release (IU/L) of coronary effluent

**Figure 4:**
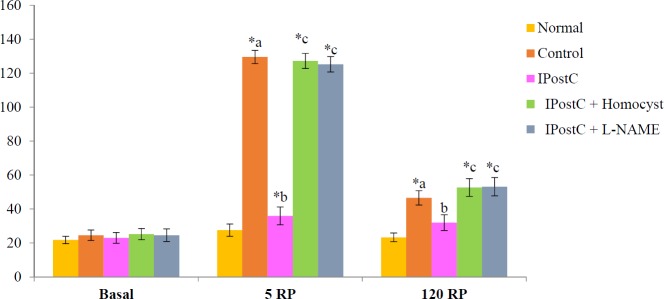
Effect of postconditioning on creatine kinase (IU/L)

Global ischaemia for 30 min. followed by reperfusion for 120 min. produced significant increase in release of LDH in coronary effluent noted 0 min,(277.00±16.71), 5 min (184.12±12.19) and 30 min. (165.00±10.05) after reperfusion. Moreover, there was significant increase in release of CK noted 5 min. after reperfusion (127.82±2.03). Ischaemic postconditioning significantly attenuated the ischaemia- reperfusion induced increase in LDH and CK. Pretreatment with LHomocysteine (300 µM), a selective inhibitor of DDAH enzyme and LNAME (100 µM) completely abolished the ischemic postconditioning induced increase in LDH and CK (Figure 3 and Figure 4).

***Effect of postconditioning on nitrite concentration***

**Figure 5:**
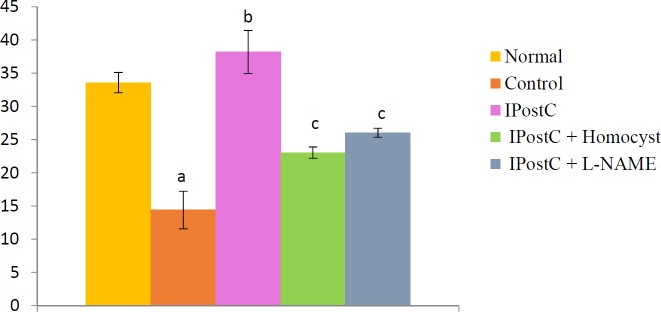
Effect of postconditioning on nitrite level (µM/L)

A significant decrease in NO (Nitrite) level was observed in tissue homogenates of hearts subjected to global ischaemia for 30 min. followed by reperfusion for 120 min. (14.44±3.85 µM/l) as compared to normal group (33.58±0.52 µM/L). Ischaemic postconditioning significantly increased the ischaemia-reperfusion induced decrease in nitrite levels.Pretreatment with L-Homocysteine (300 µM), a competitive inhibitor of DDAH and LNAME (100 µM) completely abolished the pharmacological postconditioning induced increase in nitrite concentration (Figure 5).

***Effect of postconditioning on DDAH enzyme activity***

**Figure 6:**
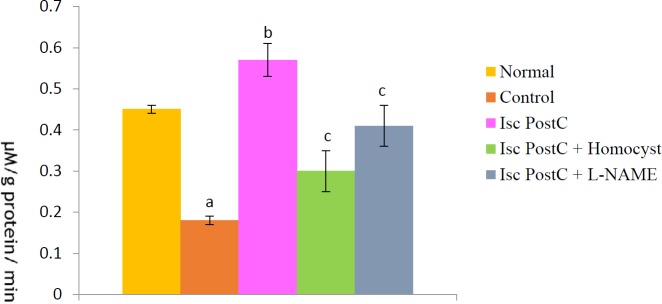
Effect of postconditioning on dimethylarginine dimethylaminohydrolase

**Asignificant decrease in DDAH enzyme activity (0.18 µM L-citruline/g protein /min) was observed in tissue homogenates of control group heartssubjected to I/R injury as compared to normal hearts (0.45 µM L-citruline/g protein /min). Ischaemic postconditioning significantly increased the ischaemia-reperfusion induced decrease in DDAH enzyme activity**Pretreatment with L-Homocysteine (300 µM), a competitive inhibitor of DDAH and LNAME (100 µM) completely abolished the pharmacological postconditioning induced increase in DDAH enzyme activity (Figure 6).

***Effect of postconditioning on ADMA level***

**Figure 7:**
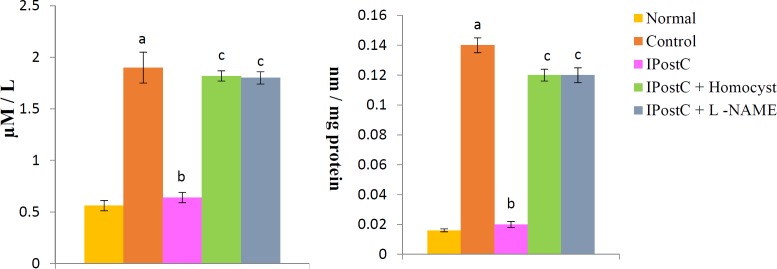
Effect of postconditioning on asymmetrical dimethylarginine level

In our study, ADMA concentrations in normal heart was 0.56 µM/L or 0.016±0.001 nm/mg. A significant increase in ADMA level (1.9±.15 µM/l) was observed in heart tissue homogenates in animals subjected to global ischemia for 30 min followed by reperfusion for 120 min. Ischaemic postconditioning significantly reduced the ischemia-reperfusion induced increase in ADMA level. Pretreatment with LHomocysteine (300 µM), a selective inhibitor of DDAH enzyme and LNAME (100 µM) completely abolished the ischemic postconditioning induced reduction in ADMA level (Figure 7).

***Effect of postconditioning on histopathological changes in myocardium***

**Figure 8:**
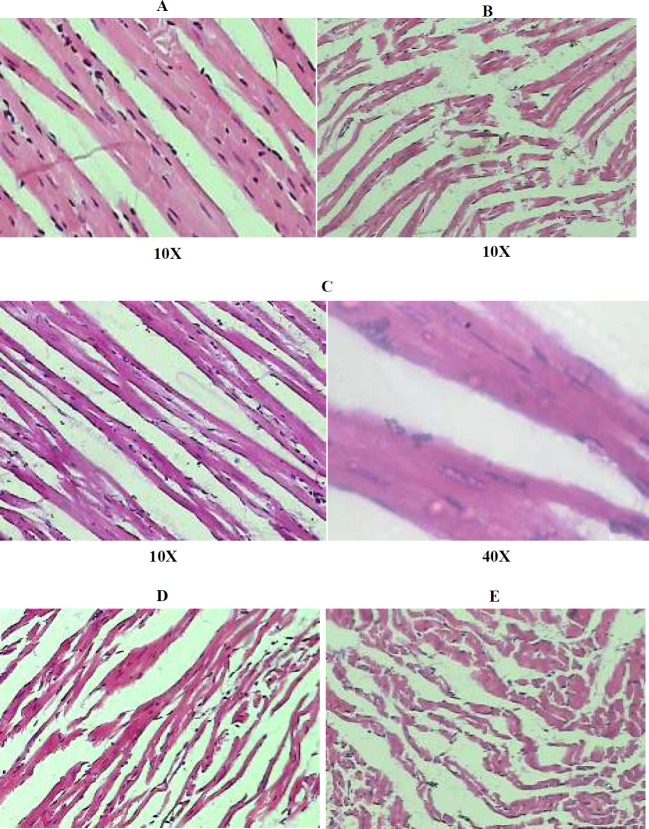
Histopathological changes in the myocardium (H & E staining)

The morphological changes are evaluated histopathologicaly by haematoxylin and eosin staining. The microphotographs of myocardium of rats subjected to global ischaemia and reperfusion revealed the myocardial membrane damage, extensive myofibrillar degeneration and infiltration of inflammatory cells as compared to those of normal group. On the other hand, in the postconditioning group, with marked improvement evidenced by the normal architecture of myofibers, reduced degree of myonecrosis, infiltration of inflammatory cells, and lesser vacuolar changes compared to the I/R group. Further these protective effects are abolishd by pretereatment with L-Homocysteine (a competitive DDAH inhibitor) and L-NAME (an e-NOS inhibitor) (Figure 8)

## Discussion

Isolated rat heart preparation perfused in retrograde manneron Langendorff apparatus^31^has been employed in the present study because it permits the use of pharmacological interventions without any interference due to changes in systemic circulation (32).Moreover, studies on isolated heart preparations have led to important discoveries in field of cardiac physiology, pharmacology and surgery (33, 34).

Infarct size was assessed macroscopically because a good correlation has been demonstrated between macroscopic and microscopic measurement of infarct size (35). Lactate dehydrogenase (LDH) and creatine kinase (CK), localized in myocytes, are released during ischemia-reperfusion induced irreversible myocardial injury. In CABG patients, it has found that CK-MB/troponin elevations in the initial 24 hr were associated with increased mortality (36). Therefore, release of these enzymes in coronary effluent is documented as an index of myocardial injury. Earlier reports from our laboratory have demonstrated that the peak release of LDH occur immediately or 5 min. and 30 min. after reperfusion, whilst peak release of CK is seen after 5 min of reperfusion during myocardial injury (37, 38). Therefore, samples of coronary effluent were collected at these time intervals to estimate the release of LDH and CK. In the present study, 30 min of ischemia followed by 120 min of reperfusion produced myocardial injury, as assessed in terms of increased infarct size in the heart and elevated release of LDH and CK in the coronary effluent.

Previous studies by Zhao *et al.* (39) reported that in a canine model of coronary artery occlusion and reperfusion, postconditioning reduced multiple manifestations of ischemia–reperfusion injury, including infarct size and endothelial dysfunction. Similarly, in the present study, an attempt has been made to examine the effect of postconditioning on ischemia reperfusion induced myocardial injury. In the present study, six cycles of postconditioning applied over the first minute of reperfusion significantly reduced infarct size, release of LDH and CK in coronary effluent as well as protected the heart against IR injury through the recovery of LVDP and ±dp/dt.

A significant decrease in nitrite level (14.4±3.80 µM/l), the stable end product of nitric oxide (NO), in heart homogenate of control group (I/R injury) as compared to normal group (33.58±0.52 µM/l) was observed. The decrease in NO generation may be due to altered eNOS activity by an endogenous NOS inhibitor, ADMA, since ADMA concentration was found to be increased in heart tissue homogenates in the animals subjected to ischemia and reperfusion injury. This increased concentration of ADMA in IR injury group can be responsible for the low bioavailability of NO. Physiologically, ADMA mediated inhibition of eNOS activity is only 10%. Under pathological conditions such as blood vessel injury, the concentration of ADMA is 3–9 times higher and eNOS activity inhibition reaches 30–70% (40).

Normally, ADMA concentration is regulated by an enzyme DDAH, which metabolizes ADMA. Many factors are able to modulate DDAH activity or change the gene expression of the enzyme. Sulfhydryl group of cysteine in its active site predisposes the enzyme for easy oxidation or nitrosation with subsequent loss of its activity (41). Various cardiovascular risk factors such as hypertension, hypercholesterolemia, hyperglycemia, and hyperhomocysteinemia and oxidative stress are the main factors affecting DDAH activity leading to the increased ADMA concentration (42, 43). It has been reported that DDAH-activity is inhibited *In vitro* by oxidative stress induced by hyperhomocysteinemia (44). Oxidative stress is also generated during first few minutes of post-ischemic reperfusion which may result in inhibition of DDAH leading to ADMA accumulation (45).

In our study, DDAH enzyme activity is inhibited after 2 hr of reperfusion which is accompanied with increase in level of ADMA and reduced NO concentration. Further, ischaemic postconditioning significantly increased the nitrite level, DDAH enzyme activity and reduced ADMA level.

Several studies have reported that fasting serum homocysteine levels are increased in coronary artery disease (CAD) patients and positively correlated with severity of CAD (46). Hyperhomocysteinemia can arise from nutritional deficiencies of folate, vitamin B6, and vitamin B12 (47, 48). A case study of a young patient with an acute myocardial infarction due to a thrombotic occlusion of the left anterior descending coronary artery who also developed pulmonary emboli in the setting of severe hyperhomocysteinemia secondary to pernicious anemia (49).

Homocysteine induced oxidative stress mediated inhibition of e-NOS supports the role of homocysteine in endothelial dysfunction (50). A study by Stulingher *et al.* has demonstrated that DDAH activity was reduced by L-Homocysteine in primary bovine aortic endothelial cells, *Ex vivo* aortic segments, as well as the activity of recombinant human DDAH in a cell free system.

In our study, we employed L-Homocysteine as competitive DDAH inhibitor to explore the role of DDAH enzyme activity in cardioprotection mediated by ischaemic postconditioning. L-Homocyteine abolished ischaemic postconditioning induced increase in nitrite level, DDAH enzyme activity and reduced ADMA level. Thus, cardioprotective effects of ischemic postconditioning may be attributed to increased DDAH activity which is associated with reduced levels of ADMA in hearts. Further reduction in ADMA level may lead to increase in activity of eNOS as demonstrated by increase in concentration of Nitric oxide (NO) or nitrite level.

Values are expressed as mean±SD. a=*P<*0.05 vs Normal; b=*P<*0.05 vs Control; c=*P<*0.05 vs Isch. PostC

**Table 1:** Effect of postconditioning on coronary flow rate (ml/min) of rats

Group	Basal	** 5RP**	**30RP**	**120RP**
Normal	8.92 ± 0.23	8.6 ± 0.36	8.42 ± 0.32	8.08 ± 0.23
Control	8.00 0.25	4.32±.42^a^	4.04 0.27^a^	2.90 0.34^a^
Ischaemic PostC	8.34 ± 0.35	8.00 ± 0.12^b^	8.14 ± 0.195^b^	7.88 ± 0.23^b^
Isch. PostC + L-Homocysteine (300 µM)	8.52 ± 0.46	3.68 ±0.48^*c^	3.92 ± 0.42^*c^	3.56 ± 0.42^*c^
Isch. PostC + L-NAME (100 µM)	8.86 0.35	4.92 ± 0.58^*c^	5.62 ± 0.71^*c^	4.54 ± 0.57^*c^

Values are expressed as mean min^-1^±SD (n=6). Basal denotes coronary flow rate (C.F.R) measured before global ischaemia. 5RP, 30RP and 120 RP denotes C.F.R measured 5, 30 and 120 min. after reperfusion following global ischaemia.*P<0.001 vs Basal; a= *P<*0.05 vs Normal; b=*P<*0.05 vs Control; c=*P<*0.05 vs Isch PostC

**Table 2:** Effect of postconditioning heart rate (beats/ min) of rat

Group	Basal	** 5RP**	**30RP**	60RP	**120RP**
Normal	220 4.87	220 5.81	218 8.37	218 8.37	214 5.48
Control (I/R Injury)	200.8 8.02	126 14.87^a^	160 11.4^a^	142 1.8^a^	76 11.60^a^
Ischaemic PostC	216.2 ± 6.49	200 7.91^b^	204 4.24^b^	206 4.55^b^	200 ± 6.24^b^
Isch. PostC + L-Homocysteine (300 µM)	208.42 ± 7.14	105.6 ± 4.56^*c^	110.62 ± 5.94^*c^	100.24 ± 5.40^*c^	92.06 ± 5.42^*c^
Isch. PostC + L-NAME (100 µM)	220.2 5.24	110 ± 4.84^*c^	120 ± 3.46^*c^	150 ± 3.26^*c^	105 ± 5.24^*c^

Values are expressed as mean±SD (n=6). Basal denotes heart rate measured before global ischaemia. 5RP, 30RP, 60RP and 120RP denotes heart rate measured 5, 30, 60 and 120 min after reperfusion following sustained global ischaemia. *P<0.001 vs Basal; a= *P<*0.05 vs Normal; b =*P<*0.05 vs Control; c=*P<*0.05 vs Isch. PostC

**Table 3:** Effect of postconditioning on left ventricular developed pressure (mm Hg) of rats

**Group**	**LVDP**	
	**Basal**	**30 RP**
Sham (Normal)	86.5 ± 4.06	86.68 ± 6.70
Control (I/R Injury)	82.67 ± 3.46	48.58 ± 3.2*^a^
Isch. PostC	85.34 ± 3.86	66.18 ± 3.46*^b^
Isch. PostC + L-Homocysteine (300 µM)	84.34 ± 5.26	45.62 ± 3.8*^c^
Isch. PostC + L-NAME (100 µM)	85.34 ± 5.86	44.64 ± 3.2*^c^

Data is represented as mean±SD. *P˂0.05 vs Basal; a= *P˂*0.05 vs Normal; b= *P˂*0.05 vs Control; c=*P˂*0.05 vs Isch. PostC

Values are expressed as mean±SD. *P<0.05 vs Basal; a=*P<*0.05 vs Normal; b=*P<*0.05 vs Control; c=*P<*0.05 vs Isch. PostC

**Table 4:** Effect of postconditioning on dp/dt max and dp/dt min

**Group**	**+dp/dt max (mmHg/s)**		**- dp/dt min (mmHg/s)**	
	**Basal**	**120 RP**	**Basal**	**120 RP**
Sham (Normal)	3105 ±120	2957±148	- 2560±210	-2270±142
Control (I/R Injury)	3093±164	1876±167	-3250± 145	-1076±140
Isch. PostC	3224±125	3310± 204	- 2983±216	-2745±178
Isch. PostC + L-Homocysteine (300 µM)	3062±154	2057±164	-2015±216	-1776±140
Isch. PostC + L-NAME (100 µM)	3073±168	2057±164	-2017±226	-1876±160

Data is represented as mean±SD. a= *P˂*0.05 vs Normal; b= *P˂*0.05 vs Control; c= *P˂*0.05 vs Isch. PostC

Values are expressed as mean±SD. *P<0.05 vs Basal; a=*P<*0.05 vs Normal; b=*P<*0.05 vs Control; c= *P<*0.05 vs Isch. PostC

Nitrite level was estimated in heart homogenate. Values are expressed as mean±SD. a=*P<*0.05 vs Normal; b=*P<*0.05 vs Control; c=*P<*0.05 vs Isch. PostC

Dimethylarginine dimethylaminohydrolase (DDAH) activity was estimated in heart homogenate. Values are expressed as mean±SD. a=*P<*0.05 vs Normal; b=*P<*0.05 vs Control; c=*P<*0.05 vs Isch. PostC

Values are expressed as mean±SD. a=*P<*0.05 vs Normal; b=*P<*0.05 vs Control; c=*P<*0.05 vs Isch. PostC

a. Microphotographs of normal group showing normal architecture of myocardial fibers with no infiltration of inflammatory cells; b. Microphotograph of control group showing cytoplasmic vaculation, infiltration of inflammatory cells and disarrangement in myocardial cell; c. Microphotographs of Ischaemic postconditioning showing normal architecture of myocardial fibers with no infiltration of inflammatory cells; d. Microphotographs of PostC + L-Homocysteine (DDAH inhibitor) (d)and PostC + L-NAME; e. showing infiltration of inflammatory cells and disarrangement in myocardial cells

## Conclusion

Therefore it may be concluded that ischaemic postconditioning inducedenhancement of DDAH activity provides a novel target to reduce ADMA level and increase NO bioavailability so as to deliver cardioprotection in myocardial ischemia-reperfusion injury. However, further studies are required to substantiate the findings of this study.
